# New photocatalytic materials based on alumina with reduced band gap: A DFT approach to study the band structure and optical properties

**DOI:** 10.1016/j.heliyon.2024.e27029

**Published:** 2024-02-28

**Authors:** Taha Yasin Ahmed, Shujahadeen B. Aziz, Elham M. A. Dannoun

**Affiliations:** a Physics Department, College of Science, University of Sulaimani, Kurdistan Regional Government, Qlyasan Street, Sulaymaniyah 46001, Iraq; b Research and Development Center, University of Sulaimani, Kurdistan Regional Government, Sulaimani 46001, Iraq; c Department of Physics, College of Science, Charmo University, Chamchamal, 46023, Sulaymaniyah, Iraq; d Department of Mathematics and Sciences, Woman Campus, Prince Sultan University, P.O. Box 66833, Riyadh 11586, Saudi Arabia

**Keywords:** α-Al_2_O_3_, DFT, Band structure & density of states, Optical properties

## Abstract

In this study, first-principles calculations using Density Functional Theory (DFT) have been conducted, which were carried out using the Vienna Ab initio Simulation Package (VASP) to examine the effect of Tl insertion on electronic and optical properties of the α-Al_2_O_3_. Alumina materials are abundant and the main shortcoming of alumina for photocatalyst applications is their large energy band gap and little absorption in the visible region of electromagnetic (EM) radiation. Insertion of transition metals (TM) into semiconductor or insulating materials is a hot approach to improve the absorption behavior of these materials using DFT assessment. In the current work an analysis of the band structure (BS) and the density of states (DOS); comprising both the total density of states (TDOS) as well as the partial density of states (PDOS) were carried out. The BS diagram revealed that various concentrations of Tl insertion into the α-Al_2_O_3_ reduced the band gap to 2.38 eV. In the density of state diagram, the band gap energy shifted to lower photon energies with increasing Tl concentrations which supports the BS results. The band gap obtained from the first peak in the imaginary part of dielectric function is close enough to those established from the BS diagram. Distinguished shifting of absorption coefficient to lower photon energy (2.27 eV) reveals the suitability of the doped α-Al_2_O_3_ for various applications. The increase of refractive index (n) with increasing of Tl into the α-Al_2_O_3_ structure is evidence for the increase of charge, which is a source for polarization and attenuates the velocity of light in a medium. The increase of optical conductivity with photon energy started after band gap values. The reflectance, absorbance and transmittance results indicate that the doped α-Al_2_O_3_ is responsive to the visible region of EM radiation while in pure state almost transparent.

## 1 Introduction

In recent years, clean energy is one of the most factors that impact the human being in terms of save life. Nowadays, renewable energy technologies have been focused accompanying with other consumable energies, for instance crude oil and fossil around the world. Two main obstacles to large-scale applications of renewable energy technologies are the low efficiency of power conversion and the cost of fabrication [1]. The greatest challenge confronting modern society is the invention of new environmentally friendly materials to substitute conventional materials [2]. Contrarily, transition aluminas are among the most valuable materials in adsorption and catalysis technologies, and they are utilized in a substantial number of key industrial processes. These materials are significant and adaptable due to the relative abundance of their surface chemistry, which connects Lewis and Brønsted acidic and basic sites [3]. Aluminum oxide (alumina) is a readily available industrial substance with tremendous practical value as an optical and catalyst material used in electroluminescence displays, optical coatings, photoelectronic machines, and stress imaging technology [4].

Alumina $\left({\text{Al}}_{2}O_{3}\right)$ is a ceramic substance of tremendous importance for both theoretical research and practical uses including durability, resistance to abrasion, corrosion resistance, mechanical strength, excellent electrical insulation, effective optical properties, small particle size, extensive surface area, and catalytic surface behavior. For example, α-${\text{Al}}_{2}O_{3}$ one of the phases of alumina, is utilized in electronics [5]. α-${\text{Al}}_{2}O_{3}$ plays a key role as a catalyst, ceramic, and dielectric material. ${\text{Al}}_{2}O_{3}$ is regarded as an appropriate insulator for a variety of electronic uses, including as a gate dielectric in metal-oxide-semiconductor (MOS) transistors and as a blocking or trapping insulator in charge-trapping nonvolatile memory cells [6]. This is owing to its broadband gap, small leakage current and mild dielectric constant value. However, it was discovered that the composite photocatalyst based on α-${\text{Al}}_{2}O_{3}$ exhibits decent photocatalytic performance for the decomposition of organic dyes because α-${\text{Al}}_{2}O_{3}$ offers a superior active adsorption site [7].

A direct band gap of 6.1 eV for γ-Al_2_O_3_, another phase of alumina, at the Γ -point was found by Samantaray et al. [8] using the Local Density Approximation (LDA) exchange-correlation potential. In contrast, Mousavi et al. [9] employed the Full Potential Linearized Augmented Plane Wave (FP-LAPW) method and identified a direct band gap of 7.2 eV for the same material. Furthermore, employing the Generalized Gradient Approximation (GGA) with the Perdew-Burke-Ernzerhof (PBE) model [10], a band gap of 6.045 eV was calculated. This value is slightly lower than the 6.26 eV band gap reported in Perevalov's study [11], likely due to pseudopotential differences. Zhao et al. [12] more recently investigated the electronic band structures of ${\text{Al}}_{2}O_{3}$ and found that it has three polymorphs, all of which are ultrawide band gap semiconductor substances with band gaps of 5.74–6.40 eV [13]. α-${\text{Al}}_{2}O_{3}$ has a broadband gap, which makes it difficult to use visible light efficiently. The broad band gap and low energy conduction band of α-${\text{Al}}_{2}O_{3}$ materials hence restrict their use. Doping or the development of hybrid materials based on α-${\text{Al}}_{2}O_{3}$ is one of the more effective ways to address the aforementioned drawbacks [14]. Recently computational materials science attracted the focus of many research groups to reduce the cost and keep the time for experimental approaches.

As a result, efforts to make it possible to use visible light are one of the hot subjects for enhancing α-${\text{Al}}_{2}O_{3}\;\text{as}\;a\;\text{photocatalyst}$, it entails increasing the absorption range of the material from UV to visible light. First-principles calculations based on Density Functional Theory (DFT) were conducted using the Vienna Ab initio Simulation Package (VASP). These calculations were utilized to investigate the structural properties, electronic band structure, and optical properties of Tl-doped α-${\text{Al}}_{2}O_{3}\text{.}$ A thallium (Tl) atom's electron configuration is [Xe] 4f^14^ 5 d^10^ 6s^2^ 6p^1^. It possesses three valence electrons in its sixth shell. It is more difficult to participate in chemical bonding than with heavier elements because of the inert pair effect, which stabilizes the electron pair in the 6s orbital through relativistic processes. Since there aren't many electrons accessible for metallic bonding, it is similar to nearby elements like lead and mercury. Consequently, Tl has a low melting point of 304 °C and is a soft, highly electrically conductive metal like its counterparts [15]. Moreover, when employing the GGA-PBE approximation for the exchange-correlation (XC) potential, there is a tendency to yield significantly underestimated energy gap values in semiconductors and insulators, with a shift towards fine-structure peculiarities near the Fermi level in their valence-band regions. Recent advancements in DFT computations suggest that the optimal agreement between experimental data and theoretical predictions for compounds containing heavy elements with 4f, 5d, and 6s electrons—such as Tl [16].

Understanding of the optical characteristics of Tl doping in α-${\text{Al}}_{2}O_{3}$ at the microscopic scale is accomplished using this method. The lattice energy of the Tl insertion at the Al substitution site can be estimated based on energy calculations. The direct band gap has been achieved following Tl doping, and pure α-Al_2_O_3_ possesses the wide energy band gap. Additionally, the valence band has shifted upward due to the confirmation of the production of new states at Γ point. Furthermore, Tl doping results in a significant band gap reduction. As a result of Tl doping in the host lattice, a red shift is seen as the optical characteristics of α-${\text{Al}}_{2}O_{3}$ are calculated.

## 2 Computational methods

**Fig. 1:**
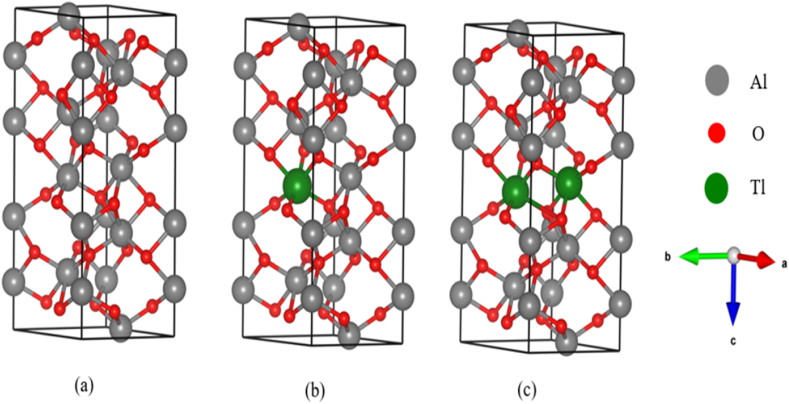
(a) α-Al_2_O_3_ unit cell for 30-atomic cell (pure system). The atoms of Al and O are colored gray and red, respectively. (b) Tl (green ball) doped α-Al_2_O_3_ by 8.33 % concentration with configuration Al_11_TlO_18_.(c) Tl (green ball) doped α-Al_2_O_3_ by 16.66 % concentration with configuration Al_10_Tl_2_O_18_. (For interpretation of the references to color in this figure legend, the reader is referred to the Web version of this article.)

The DFT calculations are carried out using Vienna Ab initio Simulation Package (VASP) and employing for exchange correlation (XC) potential the GGA approximation as developed by Perdew-Burke-Ernzerhof (PBE). All calculations use this functional, which is used to examine band structure, density of states, optical quality, and geometry optimization [17], as illustrated in Fig. 1. The α-${\text{Al}}_{2}O_{3}$ (space group R3c) unit cell for a 30 atomic cell is also shown in Fig. 1(a). This research also utilized the substitutional technique, as shown in Fig. 1 (b and c), substitutes the Tl atom for the Al atom at doping concentrations of 8.33 and 16.66 %, respectively. The structure was optimized and its properties were calculated with a 380 eV plane-wave energy cutoff. Both the pure and doped systems were analyzed using the Monkhorst-Pack technique, with k-points set at 15×15×4 [[18], [19], [20]]. For geometry optimization, the energy, energy change, force convergence, and maximum displacement tolerances were set at $2.4\times 10^{2}\;\text{eV},3.6\times 10^{-15}\;\text{eV},2.4\times 10^{2}\;\text{eV}/A^{0}$, and $1.0\times 10^{-2}A^{0}$, respectively.

## 3 Result and discussion

### 3.1 Optimized lattice parameters

Following the geometry optimization process, the lattice parameters of pure α-${\text{Al}}_{2}O_{3}$ in its hexagonal unit cell are as follows: $a=b=4.76\;A^{0}$ and $c=12.99A^{0}$ [11]. These values closely align with experimental data, showing excellent agreement with only a minimal absolute error of 0.07% [21]. Table 1 displays the structural parameters of pure α-Al2O3 from various theoretical studies. The minor deviations observed in the theoretical values are negligible. Thus, the reliability and validity of employing first-principles calculations have been confirmed.

**Table 1:** The optimized lattice parameters and direct energy bandgap values from various ab initio investigations of a pure α-Al_2_O_3_ insulator have been examined.

Functional	a, b ($A^{0}$)	c ($A^{0}$)	E_g_ (eV)	Reference
FP-LAPW(LDA) FP-LAPW(GGA) FP-LAPW(EVA)	5.128	12.992	7.2	[9]
LDA(PBE)	4.76	12.99	6.26	[11]
GGA (PBE)	4.78447	13.0684	6.045	[12]
GGA (PBE)	4.805	13.118	5.74	[22]
GGA (PBE)	4.8090	13.1264	5.937	[23]
LDA	4.7621	12.9859	6.29	[24]
GGA (PBE)	4.8211	13.1609	6.5	[25]
Heyd-Scuseria-Ernzerhof (HSE)	4.74	12.94	9.2	[26]
Exp	4.75630	12.9820	8.8	[27]
GGA (PBE)	4.76	12.99	6.47	This work

### 3.2 Band structure and density of state studies

**Fig. 2:**
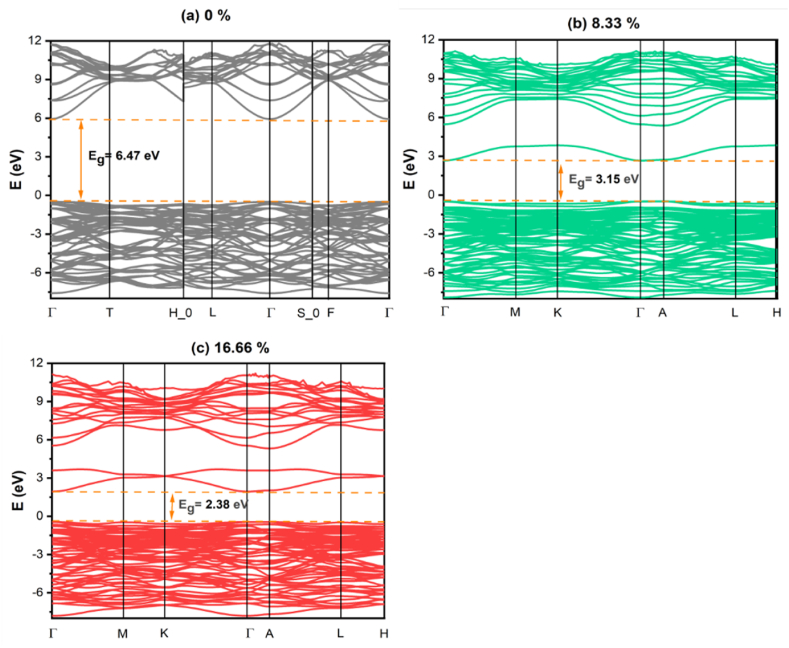
Calculated the band structure of (a) pure α-Al_2_O_3_ with (b) 8.33 % Tl doped α-Al_2_O_3_ and (c) 16.66 % Tl doped α-Al_2_O_3_ concentration. The two-orange dashed line indicate the VBM and CBm. Spin polarization is not present in this situation. (For interpretation of the references to color in this figure legend, the reader is referred to the Web version of this article.)

A material's density of states (DOS) and band structure (BS) must be adequately determined when evaluating its properties. Analyzing the electronic band structure makes it possible to ascertain the likelihood of energy ranges that electrons can inhabit, which are referred to as energy bands. In Fig. 2, an orange arrow denotes a bandgap, which is a region where electron movement is limited between the valence band maximum (VBM) and the conduction band minimum (CBM) [28,29]. The band gap shown in Fig. 2 (a) suggests that alumina is an insulator. Finding the band structure along a high-symmetry path within the Brillouin zone (Γ-T-H 0-L-Γ-S 0-F-Γ) for pure and (Γ-*M*-K-Γ-A-L-F-H) for Tl-doped α-Al_2_O_3_ was the main objective of the research conducted in this paper. For pure α-Al_2_O_3_ it can also be seen that the VBM and CBm (denoted by the orange dashed line) are located close to the high symmetry point as shown in Fig. 2(a). Moreover, aluminum (Al) has an atomic number of 13, and its electron configuration is: [Ne] 3s^2^ 3p^1^. On the other hand, oxygen (O) has an atomic number of 8, and its electron configuration is: 1s^2^ 2s^2^ 2p^4^. The conduction band displays a wide trough at Γ point, concentrated on the Al 3s orbitals. The higher valence bands consist of nonbonding oxygen p states. This demonstrates that alumina has a direct band gap, which is consistent with the majority of the outcomes [30]. As demonstrated in Fig. 2(a), the band gap calculated by GGA is 6.47 eV, which is consistent with earlier theoretical findings [24,25].

**Fig. 3:**
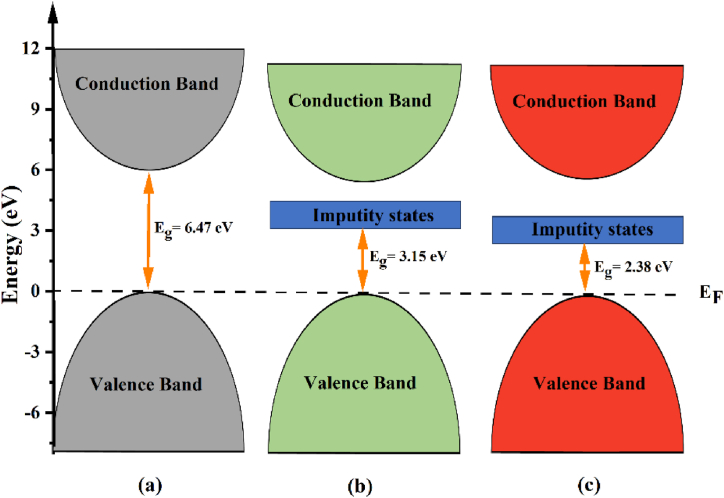
Diagram illustrating how the electronic band structure of pure α-Al_2_O_3_ (a) is impacted by the inclusion of 8.33 % Tl (b) and 16.66 % Tl (c).

In this study, we determined the band structure of pure and 8.33 % Tl and 16.66 % Tl doped α-Al_2_O_3_, as depicted in Fig. 2(b and c). This investigation sought to identify and understand the participation and characteristics of the main and dirt energy levels in the diverse Tl-doped α-Al_2_O_3_. It is clear that the band gap energy and dopant concentration are inversely related. The band gap energy decreases from 6.47 eV to 3.15 and 2.38 eV at concentrations of 8.33% and 16.66%, respectively, as illustrated in Fig. 3. This reduction is due to the fact that as described before Tl has an atomic number of 81 with an electron configuration of [Xe] 4f^14^5d^10^6s^2^6p^1^, which has valence electrons 6s^2^6p^1^ [31], which causes the impurity state to be added inside the band gap, leaving the valence band unchanged. However, the calculations also consider the core and semi-core electrons [32]. As the dopant concentration increases, it leads to more available free electrons and, consequently, a higher presence of donor states within the band gap region. Furthermore, similar to a pure system, the type of transition is direct, and the VBM and the CBM (denoted by the orange dashed line) are also located close to the high symmetry point Γ. The calculated band gap of pure and Tl-doped α-Al_2_O_3_ with different concentrations is shown in Fig. 2.

**Fig. 4:**
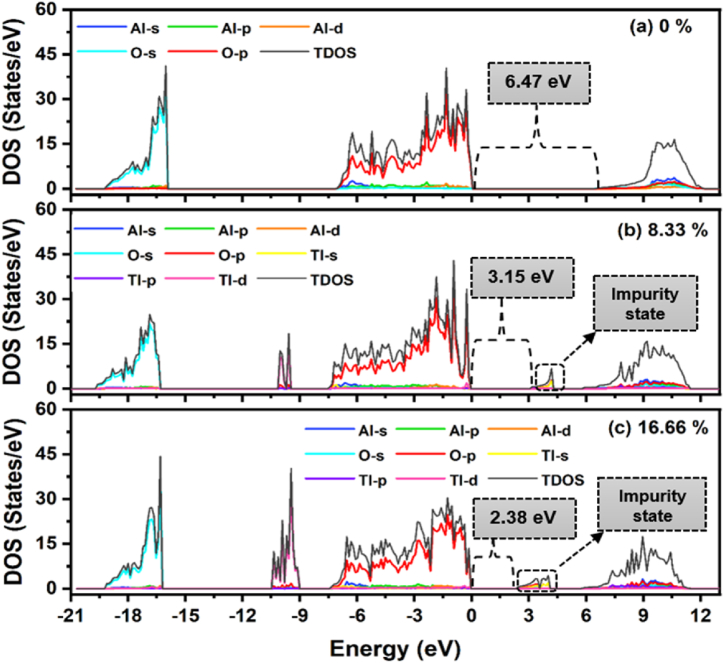
PDOS and TPOS of pure and Tl-doped α-Al_2_O_3_ with 8.33 % and 16.66 % concentrations.

To understand the impact of specific energy levels arising from the constituent atoms of pure and doped α-Al_2_O_3_, partial density of states (PDOS) and the total density of states (TDOS) were calculated, as depicted in Fig. 4. It is essential to mention that both TDOS and PDOS exhibit symmetry concerning the spin orientations associated with paired electrons. Nonetheless, unpaired electrons during doping might cause the DOS to become asymmetric. These unpaired electrons can give rise to magnetic moments [33]. However, the focus of this work does not address the intricacies of magnetic moments and magnetic characteristics. In Fig. 4(a), the PDOS and TDOS of pure α-Al_2_O_3_ are depicted. Evidently, the VBs consist of the Lower Valence Band (LVB) and the Upper Valence Band (UVB). The LVB spanning from −20 to −16 eV, mainly comprising the oxygen 2s orbital. At −17 eV, there is a sharp peak of O-2s orbitals that creates strong localized states and has a significantly higher intensity than the Al-3s orbital. While, the UVB spanning from −7 to 0 eV, primarily consisting of the oxygen 2p orbital. This observation is consistent with the conclusions of Bandura et al. [33], who previously emphasized that the oxygen 2p orbital states predominantly influence the valence band DOS in most metal oxides. Conversely, the contribution of aluminum 3s orbital is predominantly to the conduction bands, ranging from 6.47 to 12 eV. Furthermore, the energy scale of 0–6.47 eV corresponds to the band gap.

Fig. 4(b and c) shows the PDOS and the TDOS of α-Al_2_O_3_ doped with Tl at different concentrations. From Fig. 4(b) it is evident that for Tl-doped α-Al_2_O_3_ with an 8.33 % concentration, the upper valence bands segmented into two regions: spanning from −10.5 eV to −9 eV, where the majority of bands are composed of Tl-3d orbitals, accompanied by a few O-2p orbitals, and in proximity to the Fermi energy level (−7.5 eV–0 eV), where the majority of valence bands are composed of 2p orbitals of oxygen atoms, accompanied by a few 3s/3p orbitals of Al atoms. Similar to how the energy gap is reduced, the presence of impurity states, which are the Tl-6s, causes the conduction band to divide into two separate regions: an upper conduction band spanning from 5.8 to 11.5 eV and a lower conduction band spanning from 3.15 to 4.3 eV. With an increase in concentration to 16.66 %, it becomes evident that a pronounced peak emerges in the Tl-6s and O-2p orbitals, generating robust localized states. Additionally, impurity states become more pronounced, leading to a decrease in the band gap, as shown in Fig. 4(c).

### 3.3 Optical properties

The dielectric function relates the linear response of particular substances to electromagnetic radiation with respect to electromagnetic wave propagation in the medium. The electron-photon interaction, which is related to the dielectric function, is where the physical process of the inter-band transition with the solid electronic structure occurs. When an electromagnetic wave travels through a material, the relative dielectric function is represented using a complex number that considers absorption [34,35]. It is possible to write this complex number as *ε*(ω) = *ε*_1_(ω) + iε_2_(ω), where *ε*_1_(ω) and *ε*_2_(ω) stand for the real as well as the imaginary parts of the dielectric function, respectively. The possibility that energy absorbed by electrons transitions to lower energy levels, generally associated with electronic polarization or other optical processes, is taken into account by the real component of a dielectric function.

Transfer of an electron from the VB to the CB indeed causes the imaginary part of the dielectric function. This transition is responsible for energy absorption, particularly in the context of electronic excitations or optical phenomena. As a result, the ability of an electron to transition from the valence band to the conduction band depends on the dielectric function's imaginary part size. In addition, the energy scale of 1.63–3.10 eV, which corresponds to visible light, is frequently used to analyze the variation in the imaginary component of the dielectric function. This range covers the energies at which electronic transitions associated with the absorption of visible light occur in many materials. In fact, the dielectric function is a crucial aspect of a material's electronic structure and its connection to electronic transitions. It provides valuable insights into the material's band structure and optical properties, helping to understand how it interacts with light and other electromagnetic radiation. This understanding is essential for various applications, including the design of optical and electronic devices [36]. Theoretically, the momentum matrix elements of the occupied and unoccupied states can be calculated to determine the imaginary part *ε*_2_(w) of dielectric functions. Indeed, the Kramers-Kronig relationship can be used to obtain the real part of the dielectric function *ε*_1_(ω), by performing an integral over a sufficiently broad frequency range when the imaginary part, *ε*_2_(ω) is known [37,38]. Quantum physics describes the interaction of a photon with an electron in the system in terms of time-dependent perturbations of the ground electronic state. The absorption or emission of photons essentially transforms a state from having been occupied to being unoccupied. A combined DOS between the VB and conduction band (CB) is a single method to visualize the spectrum created by excitations [39]. Additionally, *ε*_1_(ω) and *ε*_2_ (ω) may be used to infer various optical properties. The optical characteristics that the density functional theory predicts are attainable, including absorption coefficient α(ω), the dielectric function *ε*(ω), refractive index n(ω), extinction coefficient k(ω) and real optical conductivity $\sigma_{r\;}\left(\omega \right)$ (equations (1), (2), (3), (4), (5), (6))), [[40], [41], [42], [43]].

(1) $$\varepsilon_{2}=\frac{C_{1}}{\omega^{2}}\sum_{V.C}\;\int_{BZ}\;d^{3}K\frac{2}{\left(2\pi \right)}{\left|e\cdot M_{CV}\left(K\right)\right|}^{2}\delta \left[E_{C}\left(K\right)-E_{V}\left(K\right)-\hbar \omega \right]$$

(2) $$\varepsilon_{1}=1+C_{2}\sum_{V.C}\;\int_{BZ}\;d^{3}K\frac{2}{\left(2\pi \right)}\frac{{\left|e\cdot M_{CV}\left(K\right)\right|}^{2}}{\left[E_{c}\left(K\right)-E_{V}\left(K\right)\right]}\frac{\hbar^{3}}{{\left[E_{C}\left(K\right)-E_{V}\left(K\right)\right]}^{2}-\hbar^{2}\omega^{2}}$$

(3) $$n\left(\omega \right)=\frac{{\left[\sqrt{\varepsilon_{1}{\left(\omega \right)}^{2}+\varepsilon_{2}{\left(\omega \right)}^{2}}+\varepsilon_{1}\left(\omega \right)\right]}^{\frac{1}{2}}}{\sqrt{2}}$$

(4) $$\alpha \left(\omega \right)=\sqrt{2}\omega {\left[\sqrt{\varepsilon_{1}^{2}\left(\omega \right)+\varepsilon_{2}^{2}\left(\omega \right)}-\varepsilon_{1}\left(\omega \right)\right]}^{\frac{1}{2}}$$

(5) $$k\left(\omega \right)=\frac{{\left[\sqrt{\varepsilon_{1}{\left(\omega \right)}^{2}+\varepsilon_{2}{\left(\omega \right)}^{2}}-\varepsilon_{1}\left(\omega \right)\right]}^{\frac{1}{2}}}{\sqrt{2}}$$

(6) $$\sigma_{r\;}\left(\omega \right)=\varepsilon_{0}\omega \varepsilon_{1}\left(\omega \right)$$

where V and C are the VB and CB, respectively, BZ is the first Brillouin zone, K, ℏ, and ω represent the electron wave vector, the Planck constant and angular frequency, respectively. The δ symbol denotes displacement, |M_CV_ (k)|^2^ refers to the momentum transition matrix elements, E_C_ (k), and E_V_ (k) stand for the corresponding energies of the CBm and the VBM, $\varepsilon_{0}$ is the static dielectric constant. Additionally, C_1_ and C_2_ are constants.

**Fig. 5:**
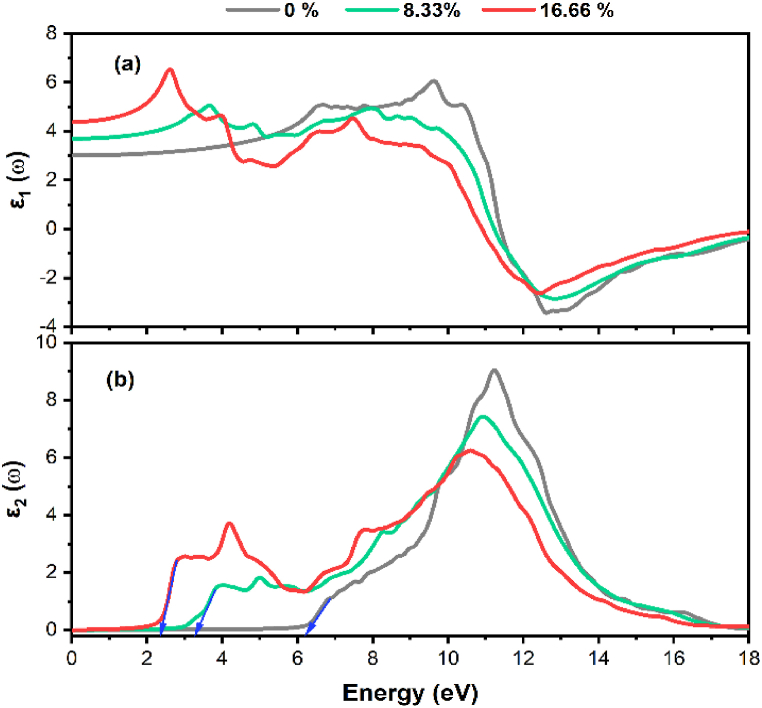
Dielectric Function component: (a) real part and (b) imaginary part, for pure and Tl-doped α-Al_2_O_3_ with 8.33 % and 16.66 % concentration.

The real component *ε*_1_(ω) of the dielectric function for pure and Tl-doped α-Al_2_O_3_ at various concentrations is depicted in Fig. 5(a). It is evident that the real part *ε*_1_(ω) increased significantly in the comparatively low energy zone, rising from 3 to 4.4. As photon energies continue to rise, the intensity of the *ε*_1_(ω) function also increases. To accurately confirm the occurrences of energy losses and inadequate light transmission through a medium with significant reflection, it is necessary to consider the real component of the dielectric constant, denoted as *ε*_1_(ω). This real component is related to the real part of the dielectric function and provides insights into how a material interacts with electromagnetic radiation, particularly in terms of absorption and energy dissipation [44]. When *ε*_1_(ω) becomes negative in a certain frequency range, it signifies that the material exhibits absorption or gain of energy in that range, which can lead to energy losses. In contrast, positive values of *ε*_1_(ω) indicate that the material is transparent to that particular range of frequencies. Therefore, by analyzing *ε*_1_(ω), you can precisely identify regions of energy losses and assess the transparency or reflectivity of the material across different frequency ranges, helping to understand its optical characteristics and performance in various applications [45]. In addition, it has been observed that the first peak of *ε*_1_(ω) has been somewhat reduced in magnitude and shifted to a lower energy region. Meanwhile, the higher energy range has noticed an increase in *ε*_1_(ω) towards positive values. These changes in the dielectric constant's behavior reflect alterations in the material's optical properties and how it interacts with electromagnetic radiation at different energy levels or frequencies.

Fig. 5(b) shows the imaginary part of the dielectric function *ε*_2_(ω) for pure and Tl-doped α-Al_2_O_3_ at different concentrations. The peaks observed in *ε*_2_(ω) represent the electronic transitions between occupied (valence) and unoccupied (conduction) states within the material. These peaks indicate the energy levels at which electrons absorb energy and move from lower energy (valence) states to higher energy (conduction) states, often associated with photons or electromagnetic radiation absorption. The positions and intensities of these peaks in *ε*_2_(ω) provide valuable information about the material's electronic structure, bandgap, and its response to different frequencies of light or other electromagnetic radiation. Analyzing *ε*_2_(ω) is a common approach in understanding the optical properties and electronic transitions within materials, especially in the context of condensed matter physics and materials science. As the illustration shows, two main peaks are evident in the spectrum of undoped α-Al_2_O_3_, occurring at 11 and 15.5 eV, respectively. The initial peak mostly corresponds to the electron transition from the upper valence band O-2p and Al-3p states to the conduction band Al-3s, Al-3p, and O-2p states. The second peak in *ε*_2_(ω) corresponds to electron transitions originating from the O-2s and Al-3s states within the valence band to unoccupied states in the conduction band.

It's interesting to note that an additional peak at 5 eV is seen when Tl-doped α-Al_2_O_3_ with 8.33 % concentration (green solid line), which is related to transitions from O-2p and Al-3p states in UVB to Tl-6s and O-2p. Notably, this extra peak contributes to the reduction of the bandgap. Furthermore, the second peak seen in pure α-Al_2_O_3_ is reduced, leading us to conclude that in this particular state, *ε*_2_(ω) features two primary peaks. The additional peak is replicated upon increasing the concentration to 16.66% (red solid line). As a consequence, *ε*_2_(ω) exhibits three main peaks. The transitions associated with the first and third peaks are analogous to those observed in the green solid line (representing doping with 8.33%).

On the other hand, the second peak results from electrons moving from the O-2p and Al-3p upper VB states to the Tl-6s state in the CB. The response of the third peak contributes in determining the band gap, much as the impact seen in the situation of 8.33% doping. Interestingly, as the amount of doping increases, the band gap reduction becomes substantial. Additionally, for Tl-doping systems, the imaginary part of the dielectric function is somewhat shifted left (by around 4.1 eV), extending the light response range. This demonstrates that some incident photons have a relatively low energy absorption efficiency in this range. More significantly, the Tl-doping system improves photocatalytic activity by increasing the imaginary part values in the high-energy region as well as the values in the visible light energy range. A crucial tool that is primarily addressed on the optical absorption spectrum. Light radiation is typically absorbed by two main processes in this class of materials, which also impact the nonmetals' transmission properties. Only at light frequencies close to the relaxation frequency of the component atoms is absorption by electronic polarization essential. The second approach depends on electron transitions from the VB to the CB, which are governed by the electron energy band structure of the material, which varies for insulators and semiconductors.

**Fig. 6:**
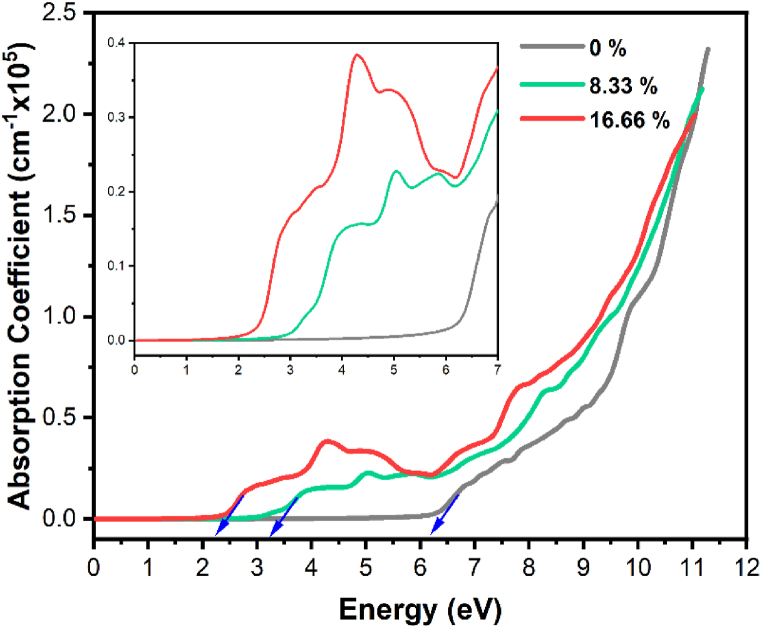
Absorption coefficient of pure and Tl-doped α-Al_2_O_3_ with 8.33 % and 16.66 % concentrations.

Fig. 6 depicts the absorption coefficients of both pure and Tl-doped α-Al_2_O_3_ at various concentrations. The absorption pattern in the doped system exhibits a pronounced red shift. As a result of the band gap reducing during this red shift, the absorption edge shifts toward lower energy levels. The observed responses of the peaks are consistent with electron transitions from the O-2p and Al-3p states to various other electronic states within the material, including the Al-3s, Al-3p, and O-2p states. The estimated absorption edge value for the pure system is 6.2 eV, however there is a noticeable red shift in Tl-doped α-Al_2_O_3_, which denotes a trend towards lower energy levels. The sharp rise in the edge becomes more pronounced with increasing photon energies, exposing strong absorption up to approximately 11 eV. Within this energy range, the alpha function exhibits several distinct fine-structure characteristics attributed to various electronic transitions [46].

Several phenomena occur as light transfers from one medium to another, such as from air to a solid. At the interface between two media, a portion of the light radiation is reflected while the remainder is absorbed or taken up by the medium. The overall intensities of the transmitted beam (I_T_), the reflected beam (I_R_), and the absorbed beam (I_A_) must be identical to the initial intensity (I_0_) of the light beam that reaches the surface of the solid medium. The relationship can be stated as follows:

(7) $$I_{0}=I_{T}+I_{R}+I_{A}$$

The quantity of energy being transmitted through a unit area per unit of time, typically measured perpendicularly to the direction of propagation, is known as radiation intensity, expressed in watts per square meter. Alternatively, Eq. (7) could be represented as follows:

(8) T+R+A=1

where T, A, and R stand for the corresponding values of transmissivity (I_T_/I_0_), absorptivity (I_A_/I_0_), and reflectivity (I_R_/I_0_). The numbers show how much incident light an object transmits, absorbs, and reflects. Since every incident light can either be reflected, absorbed, or transmitted, their sum must be equal to one. A quickly varying electric field is one element of an electromagnetic wave. With each shift in the electric field component's direction for the visible range of frequencies, the interaction between this electric field and the electron cloud surrounding each atom in its path results in electronic polarization or shifts of the electron cloud with respect to the atom's nucleus. Two events happen due to this polarization: (1) some of the radiation energy may be absorbed, and (2) the speed of the light waves as they travel through the medium slows down. The second phenomenon is refraction. Given that electronic polarization results in the slowing of electromagnetic radiation in a medium, the size of the atoms or ions that make up the medium substantially influences the amount of this phenomenon. In general, larger atoms or ions tend to exhibit more substantial electronic polarization, leading to a reduction in velocity and a subsequent increase in the index of refraction.

**Fig. 7:**
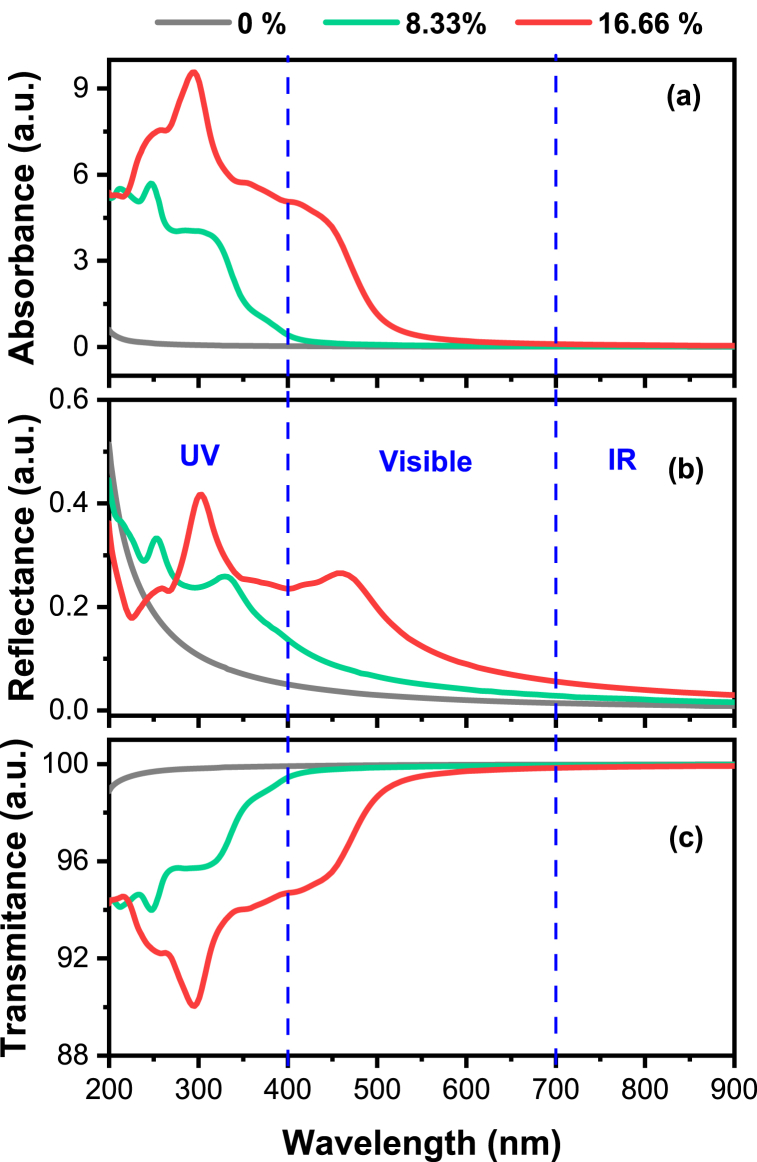
(a) Absorbance, (b) reflectance and (c) transmittance of pure and Tl-doped α-Al_2_O_3_ with 8.33 % and 16.66 % concentration.

Fig. 7 shows that (a) absorbance, (b) reflectance, and (c) transmittance of pure and Tl-doped α-Al_2_O_3_ with different concentrations. Indeed, pure α-Al_2_O_3_ lacks absorption within the visible and infrared (IR) regions. However, it demonstrates effective absorption in the ultraviolet (UV) region. On the contrary, Tl-doped α-Al_2_O_3_ displays a mild absorption within the visible spectrum and is devoid of absorption in the infrared region as shown in Fig. 7(a). Absolutely, as the doping concentration increases, there's a potential for the absorption edge to shift towards the infrared (IR) region. This shift occurs due to changes in the material's electronic structure caused by the presence of dopant atoms. Higher doping concentrations can modify the band structure, resulting in the absorption edge moving to lower energy levels, thus extending into the IR spectrum. This phenomenon is commonly observed in doped materials and can significantly affect their optical and electronic properties. As a commonly understood fact, the energy range of visible light spans between 1.62 eV and 3.11 eV (400 nm and 700 nm). The heightened absorption observed in the visible and near-ultraviolet spectrum arises from electronic interband transitions within the Tl-3d states. The “scissors operator” was used to make a systematic assessment of the UV–Vis absorption spectra for these systems moreover to the investigation of the dielectric function, as shown in Fig. 7(a). According to earlier studies, pure α-Al2O3 can only absorb ultraviolet light because of its intrinsically wide band gap.

This feature emphasizes the narrow range of applications for pure α-Al_2_O_3_. After Tl doping, the electronic structural composition changes, leading the optical absorption edge to extend into the visible light area. This expanded absorption edge now covers a wavelength range of 450–500 nm, corresponding to energy levels between 2.49 eV and 2.76 eV. As a result, Tl-doped α-Al_2_O_3_ absorbs visible light more effectively than pure α-Al_2_O_3_. A narrower band gap, which in turn reduces the energy required for an electron transfer, is associated with the red-shift phenomenon. There is a slightly greater absorption of visible light in the case of 16.66% Tl-doping because the band gap has been somewhat narrowed compared to a concentration of 8.33% Tl-doping. The impurity states are also located close to the valence band's upper section. By utilizing this property, the photocatalytic activity of materials can be increased by capturing photo-generated holes. When impurity states are present, they interact concurrently with the lower edge of the CB and the upper edge of the VB. The smallest band gap, measuring 2.3 eV, is created as a consequence of this interaction. As a result, the optical absorption edge broadens dramatically and even enters into the neighboring infrared range. This unique occurrence raises the possibility of using Tl-doped α-Al_2_O_3_ as a promising photocatalytic material, providing an alternative to TiO_2_, especially in the visible light spectrum.

The complex refractive index encompasses two components: the refractive index (n(ω)) and the extinction coefficient (k(ω)), both of which vary with energy (frequency). The refractive index serves as a gauge of a material's transparency, indicating how much light is slowed down when passing through the material. On the other hand, the extinction coefficient measures the attenuation within the material caused by the interaction of electromagnetic waves, indicating how much the light's intensity is reduced due to absorption and scattering as it traverses the material [47]. These two properties provide valuable insights into how light interacts with and passes through a given material. In addition, a material's refractive index is characterized as the ratio of the speed of light in a vacuum (c) to the speed of light within the medium. The value of (n(ω)), which signifies the extent of bending of light, is contingent on the wavelength of the light. Different wavelengths of light interact with materials in distinct ways, leading to variations in the refractive index and subsequently affecting the path and direction of light as it passes through the medium. Nonmetallic materials, owing to their electron energy band structures, can exhibit transparency to visible light. As a result, it is also essential to account for refraction and transmission phenomena alongside reflection and absorption. As described before, when light penetrates the inner regions of transparent materials, it undergoes a reduction in velocity, causing it to change direction at the interface. This phenomenon is known as refraction. In order to quantify the level of interaction between atoms and light, it's necessary to ascertain both the refractive index and the extinction coefficient. These properties are closely tied to the local polarizability and density of the materials. The n(ω) and k(ω) can be acquired through Eqs. (3) and (5), respectively [40]. Moreover, a connection exists between the refractive index and the dielectric constant for transparent materials. As previously explained, refraction is linked to electronic polarization at higher frequencies, particularly within the visible light range. As a result, it's possible to deduce the electronic component of the dielectric constant through measurements of the refractive index. This relationship between refractive index and dielectric constant helps provide insights into the behavior of materials when subjected to electromagnetic fields, particularly in the context of light interactions.

**Fig. 8:**
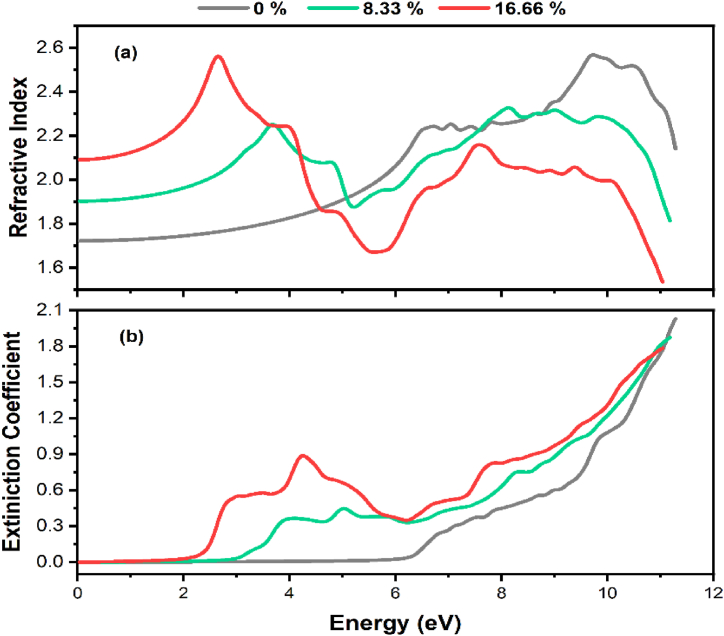
(a) Refractive index and (b) extinction coefficient of pure and Tl-doped α-Al_2_O_3_ with 8.33 % and 16.66 % concentration.

The refractive index and extinction coefficient of pure and Tl-doped α-Al_2_O_3_ in various concentrations shown in Fig. 8. The refractive index inside the visible range improves as the Tl concentration rises, as illustrated in Fig. 8(a). The noticed rise in refractive index after doping supports the idea that α-Al_2_O_3_ changes from an insulating material to a semiconductor material when Tl is added. This change in refractive index denotes modifications in the material's optical and electronic properties due to the addition of Tl dopants, denoting a transition in the material's behavior from an insulator to a semiconductor. Increased dopant concentrations may modify how light interacts with the substance, increasing the refractive index in the visible spectrum. However, it should be noted that the refractive index exhibits a plateau-like characteristic in the infrared (IR) region, where it tends to stay relatively constant.

On the other hand, the refractive index of the doped systems slightly decreases and shows variations in the UV area (3.1–6 eV). This pattern might be explained by the complex interplay of electronic transitions and interactions introduced by the dopant atoms within this energy range. This can be attributed to the behavior of electrons in deep level orbitals, which are shielded by electrons in shallower orbitals during transitions. As a consequence, electrons in deep level orbitals encounter more resistance when transitioning, leading to a reduction in the refractive index for this specific range. When a system is exposed to light of a certain energy, the extinction coefficient, which is directly related to the absorption spectrum, indicates the dissipation or absorption of energy inside the system. Indeed, k(ω) can be used to deduce the systems' energy band gap. By analyzing the behavior of k(ω) and identifying notable changes or transitions, researchers can extract valuable insights into the energy band gap characteristics of the studied systems. In essence, it quantifies the degree of light absorption within α-Al_2_O_3_. The enhancement observed in the extinction coefficient can be attributed to the states induced by Tl doping, as illustrated in Fig. 8(b). These induced states play a role in modifying the material's absorption behavior, aligning with the band structure and absorption coefficient findings. This consistency among various results underscores the influence of Tl doping on the absorption properties of the material.

**Fig. 9:**
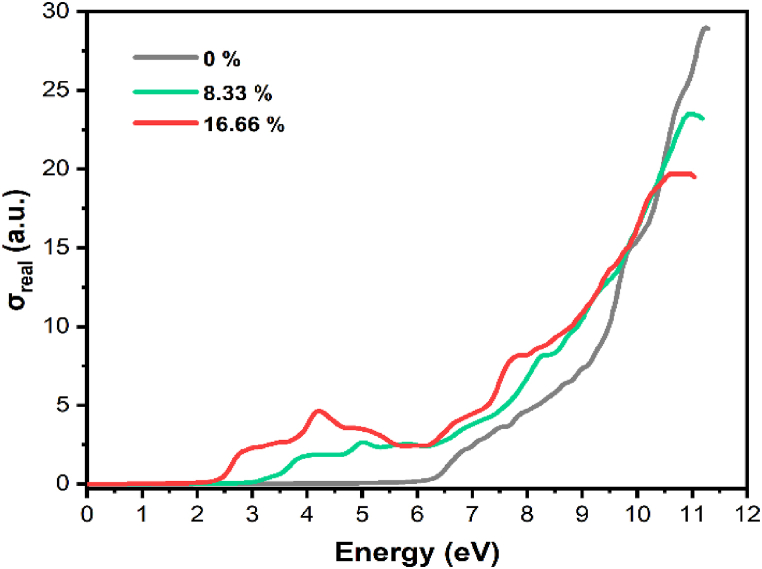
Optical conductivity of pure and Tl-doped α-Al2O3 with 8.33 % and 16.66 % concentration.

As depicted in Fig. 9, the optical conductivity demonstrates changes with respect to the incident photon energy. There's a noticeable increase in optical conductivity as the photon energy rises, particularly in the case of pure α-Al_2_O_3_. This heightened optical conductivity at higher photon energies can be attributed to the material's increased transmittance, suggesting that it allows light to pass through at those energy levels more effectively. Additionally, as the dopant concentrations increase, there is a tendency for the optical conductivity also to increase. The changes in electrical structure and optical characteristics brought about by the Tl atoms are to blame for this phenomenon. Higher dopant concentrations can change the material's ability to conduct and interact with light, resulting in an overall rise in optical conductivity.

## 4 Conclusions

In this study, we used first-principles calculations using DFT implemented in VASP to examine the electronic and optical characteristics of neat α-Al2O3 and Tl-doped α-Al2O3 at the microscopic scale. Based on energy calculations, we were able to quantify the lattice energy associated with the incorporation of Tl at the Al replacement site. The BS diagram demonstrated that introducing various concentrations of Tl into α-Al_2_O_3_ decreased the energy band gap (EBG) from the 6.47 eV–3.15 eV and 2.38 eV at Tl concentrations of 8.33% and 16.66%, respectively. In addition, the type of transition was direct in the pure and dopant systems. In the density of state diagram, it was manifested that the EBG shifted noticeably due to the emergence of impurity states. As the dopant concentration increased, a larger group of available free electrons attributed to impurity will diffuse into the donor states in the band gap region. The real part of the optical dielectric function increased in the relatively low-energy region, from its initial value of 3–4.4. It is significance mentioning to notice that the EBG from the imaginary component of the dielectric function is quite nearly matched the EBG seen in the BS diagram. The absorption coefficient was shifted to lower photon energy from 6.2 to 2.2 eV for a 16.66% concentration. The structural, electronic, and optical properties indicate that doped α-Al_2_O_3_ can be a photocatalytic material due to wide absorption in the visible region of EM radiation. The refractive index (n) increased with increased dopant concentration from 1.71 to 2.1 for maximum concentration, which is evidence for the increased charge, which is a source for polarization and attenuates the velocity of light in a medium. The reflectance, absorbance, and transmittance results indicated that the doped α-Al_2_O_3_ is responsive to the visible region of EM radiation, while in its pure state it is almost transparent. Moreover, the optical conductivity increased with increased photon energy and was found to start after band gap values.

## Data availability

Data associated with this study has not been deposited into any publicly repository. All data relevant to this study has included in article. For any further data necessities beyond what is presented, we are more than willing to supply it upon request.

## CRediT authorship contribution statement

**Taha Yasin Ahmed:** Writing – original draft, Software, Methodology, Formal analysis, Conceptualization. **Shujahadeen B. Aziz:** Writing – review & editing, Supervision, Project administration, Conceptualization. **Elham M. A. Dannoun:** Writing – review & editing, Validation, Project administration, Funding acquisition, Conceptualization.

## Declaration of competing interest

The authors declare that they have no known competing financial interests or personal relationships that could have appeared to influence the work reported in this paper.
